# Regulation of Ethanol-Related Behavior and Ethanol Metabolism by the Corazonin Neurons and Corazonin Receptor in *Drosophila melanogaster*

**DOI:** 10.1371/journal.pone.0087062

**Published:** 2014-01-28

**Authors:** Kai Sha, Seung-Hoon Choi, Jeongdae Im, Gyunghee G. Lee, Frank Loeffler, Jae H. Park

**Affiliations:** 1 Department of Biochemistry and Cellular and Molecular Biology, University of Tennessee, Knoxville, Tennessee, United States of America; 2 Department of Microbiology, University of Tennessee, Knoxville, Tennessee, United States of America; 3 Genome Science Technology, University of Tennessee, Knoxville, Tennessee, United States of America; Wake Forest University, United States of America

## Abstract

Impaired ethanol metabolism can lead to various alcohol-related health problems. Key enzymes in ethanol metabolism are alcohol dehydrogenase (ADH) and aldehyde dehydrogenase (ALDH); however, neuroendocrine pathways that regulate the activities of these enzymes are largely unexplored. Here we identified a neuroendocrine system involving Corazonin (Crz) neuropeptide and its receptor (CrzR) as important physiological regulators of ethanol metabolism in *Drosophila*. Crz-cell deficient (Crz-CD) flies displayed significantly delayed recovery from ethanol-induced sedation that we refer to as hangover-like phenotype. Newly generated mutant lacking Crz Receptor (*CrzR^01^*) and *CrzR*-knockdown flies showed even more severe hangover-like phenotype, which is causally associated with fast accumulation of acetaldehyde in the *CrzR^01^* mutant following ethanol exposure. Higher levels of acetaldehyde are likely due to 30% reduced ALDH activity in the mutants. Moreover, increased ADH activity was found in the *CrzR^01^* mutant, but not in the *Crz-CD* flies. Quantitative RT-PCR revealed transcriptional upregulation of *Adh* gene in the *CrzR^01^*. Transgenic inhibition of cyclic AMP-dependent protein kinase (PKA) also results in significantly increased ADH activity and *Adh* mRNA levels, indicating PKA-dependent transcriptional regulation of *Adh* by CrzR. Furthermore, inhibition of PKA or cAMP response element binding protein (CREB) in CrzR cells leads to comparable hangover-like phenotype to the *CrzR^01^* mutant. These findings suggest that CrzR-associated signaling pathway is critical for ethanol detoxification via Crz-dependent regulation of ALDH activity and Crz-independent transcriptional regulation of ADH. Our study provides new insights into the neuroendocrine-associated ethanol-related behavior and metabolism.

## Introduction

Chronic ethanol consumption causes serious health problems such as liver cirrhosis and various types of cancer [1]. Detoxification of the ethanol involves a sequence of reactions, in which ethanol is first oxidized to acetaldehyde by ADH, and then further oxidized to acetate by mitochondrial ALDH. Acetaldehyde promotes adduct formation, leading to the dysfunction of various key proteins and DNA damage [2]. Thus, the accumulation of acetaldehyde introduces significant toxic effects, causing the ethanol-associated health problems. Therefore, the regulatory mechanisms of ethanol metabolism are important to understand pathophysiological effects of ethanol.

The rate of ethanol metabolism in individuals is greatly influenced by genetic polymorphisms, which gives rise to enzyme variants with different catalytic properties [3], [4]. In addition to the genetic factors, transcriptional regulation and post-translational modifications affect ADH/ALDH activity [5]–[8]. Interestingly, hormones, such as thyroid and growth hormone, modulate ADH activity and its expression, indicating that hormonal factors could play a part in the ethanol detoxification [9]–[13]. However, little is known about neuroendocrine regulation of ethanol metabolism.

The genetic basis of ethanol-induced behaviors has been investigated in the fruit fly, *Drosophila melanogaster*, since its progressive behavioral patterns in response to acute ethanol exposure are quite similar to those of humans [14]. Moreover, genetic and transgenic toolkits available for this species make it an attractive model system to investigate the molecular mechanisms underlying pathophysiological and behavioral outcome of the ethanol consumption in vertebrates. Various studies have reported that several neuropeptides are involved in the regulation of ethanol-related behavior in *Drosophila* [15]–[18]. These studies suggest that peptidergic networks play an important role in modulating sensitivity to ethanol.

The neuropeptide Corazonin (Crz) was first found in the American cockroach [19]. Although the sequence and structure of Crz is highly conserved among different insect species [20], it has been shown to affect diverse physiological functions in a species-specific manner; cardio-acceleration in the cockroach [19], induction of cuticular pigmentation in the migratory locust [21], reduction of the spinning rate and pupal development in the silkworm [22], and induction of ecdysis in a moth [23]. In *Drosophila* adult, Crz is produced by a major group of neurosecretory cells in the brain and abdominal ganglion [24], [25]. Given the complexity of the Crz neuronal architecture, Crz is predicted to deliver multiple biological functions: Crz neurons are shown to be associated with sperm transfer and copulation duration [26], regulation of trehalose levels [25], and responses to physiological and nutritional stresses [27], [28].

*Drosophila* CrzR is a member of G-protein coupled receptor (GPCR) family and structurally homologous to the mammalian GnRH (Gonadotropin releasing hormone) receptor [29]. Physiological roles of the CrzR have not been explored. Using mutant flies lacking Crz neurons or CrzR, we investigated biological functions of the Crz signaling system in *D. melanogaster*. Both types of mutant flies displayed significantly delayed recovery from ethanol-induced sedation. We further show that such ‘hangover-like’ phenotype of the *CrzR* mutant likely results from fast acetaldehyde accumulation due to higher ADH production as a result of transcriptional up-regulation and lower ALDH activity. Only the latter event was observed in the Crz-CD mutant, suggesting a complicated signaling mechanism associated with the CrzR.

## Results

### Hangover-like phenotype mediated by *Crz* neurons

To explore the role of the Crz signaling pathway in ethanol-induced behavior, we employed Crz-cell deficient (Crz-CD) *D. melanogaster* that was produced by transgenic expression of a cell death gene *hid*. When flies were exposed to the vapor derived from 30–70% ethanol, we did not see a significant difference in the rates of sedation between controls and Crz-CD [30]. This is in contrast to a recent report that showed that Crz-CD is resistant to ethanol-induced sedation [16]. We do not know the cause of such discrepancy, except for the experimental setting.

**Figure 1:**
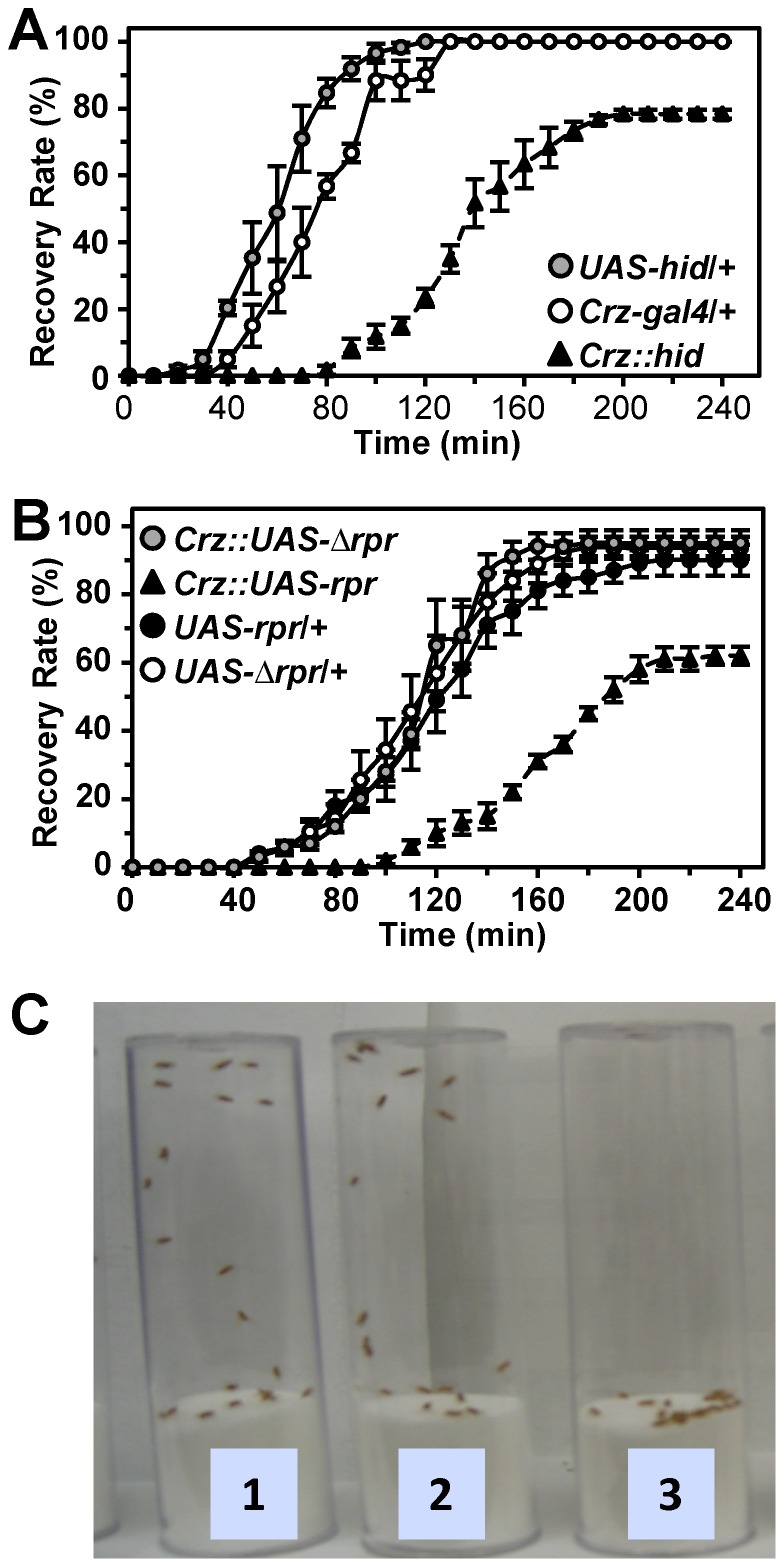
Hangover-like phenotype of Crz-CD. Crz neuron ablation induced by *hid* (A) and *rpr* (B) transgene expression (triangles) leads to delayed and incomplete recovery compared to wild-type (circles). Each data point is a mean ± sem (n = 3–5). All genotypes are in *y w* background. (C) Flies recovered from ethanol-induced sedation at 2 hours after exposure. (1, Crz-gal4/+; 2, UAS-hid/+; 3, Crz-gal4/UAS-hid).

During this study, however, we noticed that Crz-CD flies showed significantly delayed recovery from ethanol-induced sedation. To characterize this phenotype further, flies that were completely sedated with vapor from 100% ethanol for 17∼18 min were allowed to recover in an ethanol-free environment. Control flies began to assume normal standing posture and mobile around 40 min and most of them recovered after 2 h. In contrast, the recovery of Crz-CD flies (*Crz::hid*) was significantly delayed; it was first observed at 80 min and maximum 80% of flies recovered by 4 h while the remaining flies did not survive (Figure 1, A and C). Crz-CD flies generated by ectopic expression of a different cell death gene *reaper* (*rpr*) produced results similar to the *Crz::hid*, whereas expression of a mutant *rpr* (*▵rpr*, an *rpr* lacking IBM death domain) showed no difference from controls (Figure 1B). These results suggest that *Crz* neurons are required for the recovery from ethanol-induced sedation. We refer to such delayed recovery from ethanol-induced sedation as “hangover-like” phenotype.

### Severe hangover-like phenotype of a *CrzR* null mutant

**Figure 2:**
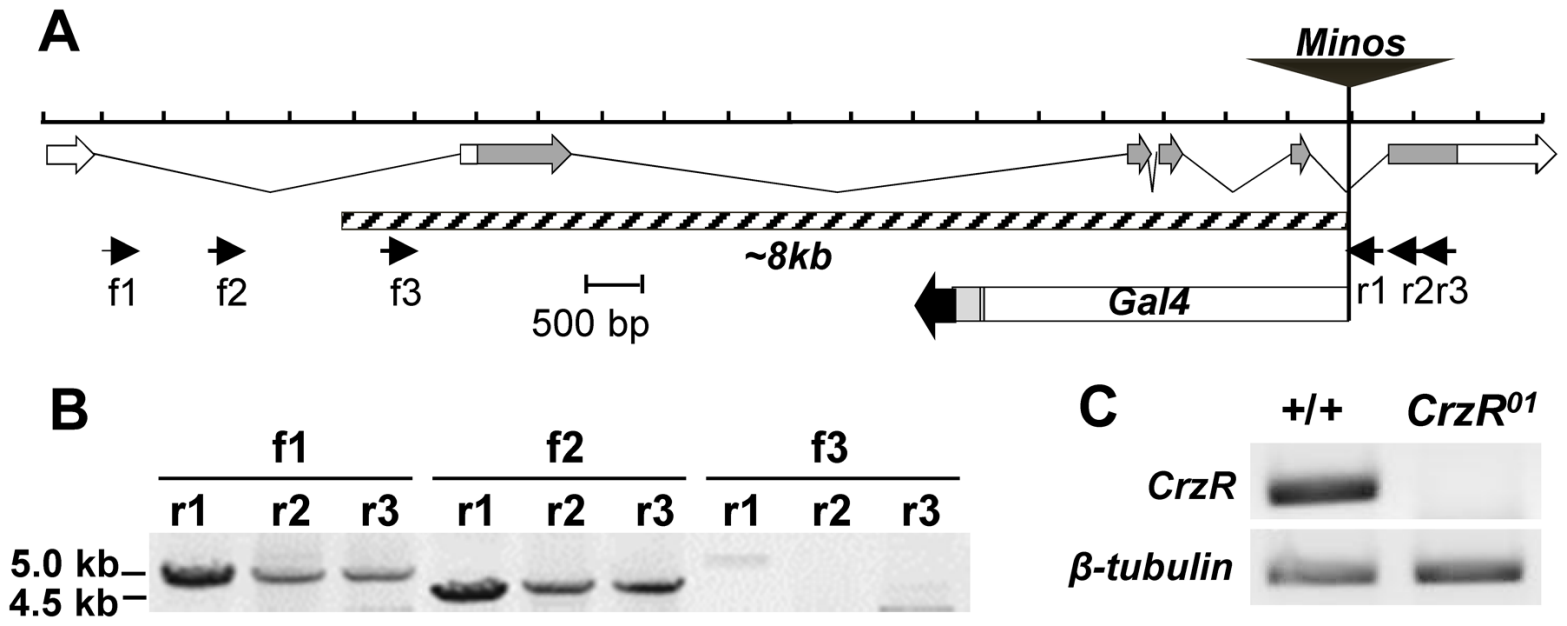
Generation of *CrzR*-null allele. (A) Diagram of *CrzR* encoding exons and approximate locations of PCR primers. Exons and introns are shown as arrows and solid lines, respectively. Coding exons are shown in grey, and UTRs in white. An 8-kb deletion was indicated by the hatched box. A footprint of *gal4* coding region and flanking sequence derived from the *Minos* element was found at the original insertion site. (B) PCR products derived from the designated primer sets and mutant genomic DNA. No specific PCR product was produced from f3 primer. (C) RT-PCR. *CrzR^01^* did not produce PCR product, while the wild-type did it with expected size.

To investigate whether the foregoing result is due to the lack of Crz signaling, we generated a null mutant lacking *CrzR* (a.k.a. *GRHRII*). A putative hypomorphic mutant allele, *Mi{ET1}GRHRII^MB00838^* (for short, *MB00838*), carries a *Minos* transposable element bearing eye-specific GFP (green fluorescence protein) marker inserted in the fifth intron (Flybase; Figure 2A). To generate a *CrzR-*null mutant, we mobilized *MB00838* using heat-shock-induced expression of *Minos* transposase [31]. The mobilization was confirmed by a mosaic pattern of GFP expression in the eyes. Subsequently, we established one hundred GFP-negative lines, one of which was identified to be a deletion mutant lacking *CrzR* by PCR. Further refined PCR confirmed an 8-kb deletion including exon 2–5 (Figure 2). In addition to this deletion, PCR revealed a footprint of the *gal4*-coding region derived from the *MB00838* element at its original insertion site. RT-PCR targeting the 5' region of *CrzR* (–59∼+328) confirmed the absence of *CrzR* transcription, suggesting that this is a *bona fide* null mutant (Figure 2C). Thus we designate this mutant as *CrzR^01^* for the first amorphic allele of this gene.

Homozygous *CrzR^01^* mutants were viable and fertile and had no discernible morphological or developmental aberrations. Mutant females are reproductively normal as their fecundity is comparable to wild-type (Figure S1). In addition, circadian locomotor activity rhythms are indistinguishable between wild-type and *CrzR^01^* mutant flies, indicating that *CrzR^01^* mutants are as healthy as wild-type (Figure S2).

**Figure 3:**
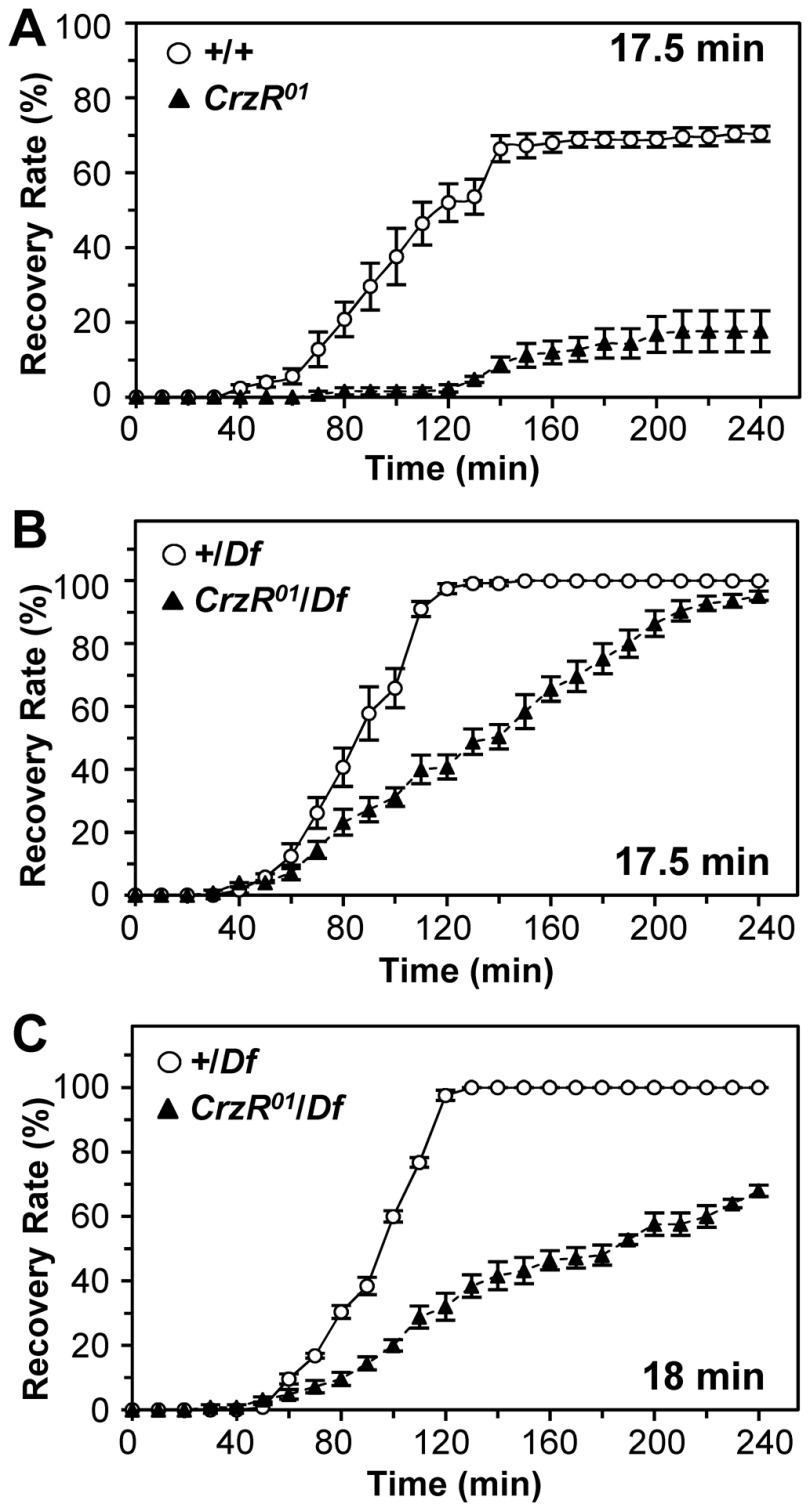
Hangover-like phynotype of the *CrzR^01^* mutant flies. Flies were exposed to 100% ethanol as indicated. (A) Homozygous *CrzR^01^.* (genotypes: *y w;; CrzR^01^*) (B) *CrzR^01^*/*Df*. (genotypes: *w^1118^*;; *+/Df*: *w^1118^*;; *CrzR^01^*/*Df*). (C) Same as in (B), except for 18-min exposure period. Each data point represents mean ± sem (n = 5).

Next we measured hangover-like phenotype of the *CrzR^01^* mutant, as done for Crz-CD. When flies were exposed to ethanol for 17.5 min, the recovery of *CrzR^01^* flies was severely retarded; only 20% recovered fully after 4 h, whereas 70% of control flies did (Figure 3A). To confirm whether this is caused by the deletion of the *CrzR* locus, we performed similar assays using hemizygous *CrzR^01^* (*CrzR^01^*/*Df*) or control (+/*Df*) with *w^1118^* background. After 17.5-min exposure, most *Df/+* flies recovered after 2 h, while *CrzR^01^/Df* did after 4 h (Figure 3B). The recovery of *Df/+* flies was largely unaffected when the exposure duration was increased to 18 min, while only 70% of *CrzR^01^*/*Df* recovered by 4 h (Figure 3C). Similar hangover-like phenotype was observed with RNA interference (RNAi)-mediated *CrzR* knock-down flies (*CrzR*-KD). *CrzR^RNAi^* was constructed as described in the supporting protocol (Protocol S1). In response to an *actin-gal4*, two independent lines, *UAS-CrzR^RNAiS3S^* and *UAS-CrzR^RNAiT17^*, showed severe reduction of *CrzR* mRNA (Figure S3A). These *CrzR-*KD flies displayed hangover-like phenotype, with around 20% and 35% of the flies recovered by 4 h, which is in contrast to 80–90% recovery of the heterozygous transgenic controls (Figure S3B). Taking together with the data obtained with Crz-CD flies suggests that Crz signaling is causally associated with the hangover-like phenotype. This phenotype is unlikely to represent a general response to sedative agents, as neither Crz-CD nor a *CrzR^01^* mutation affected the recovery rates from ethyl ether-induced sedation (Figure S4).

### Defective acetaldehyde metabolism in *CrzR^01^* mutant

**Figure 4:**
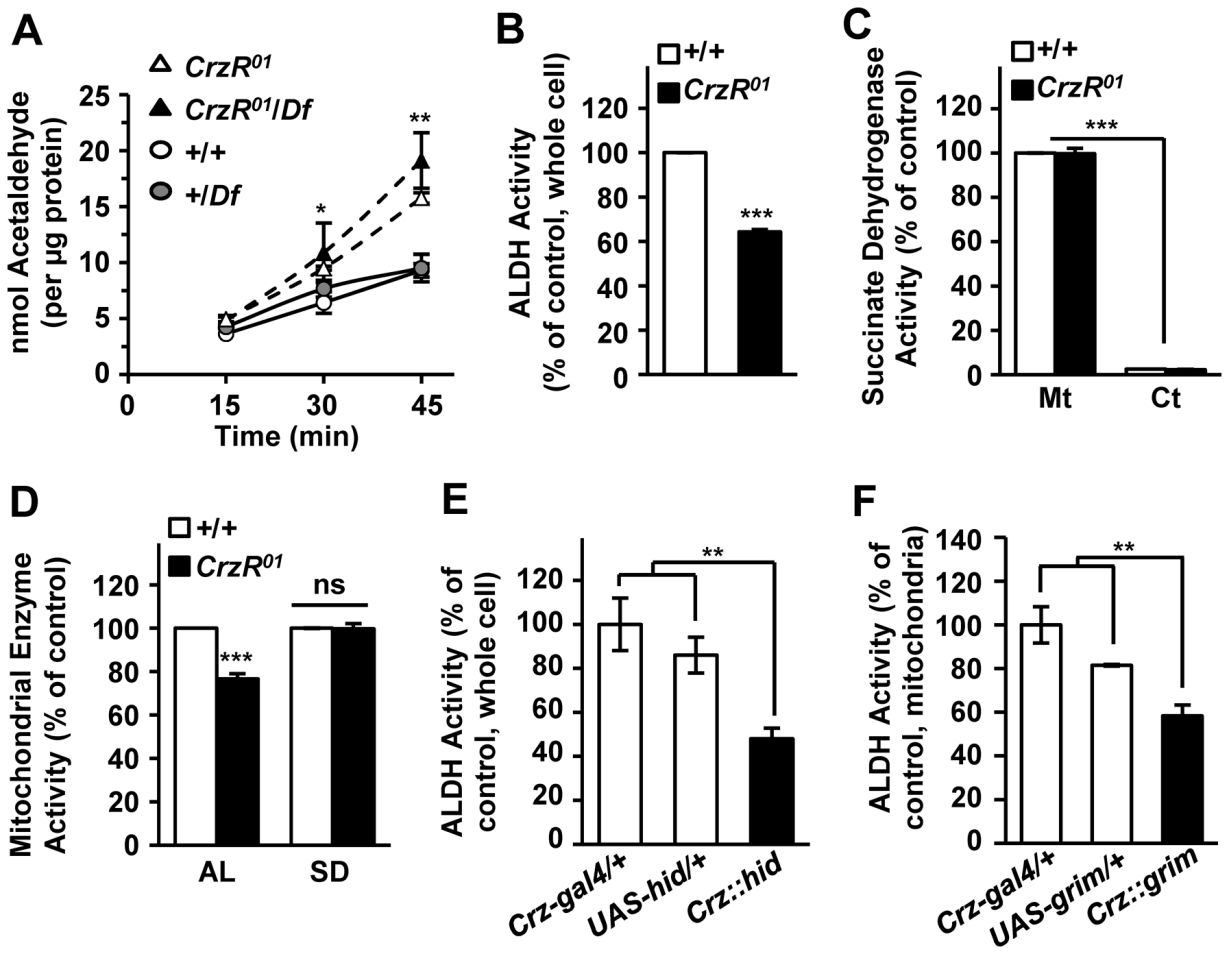
Lack of *Crz*/*CrzR* leads to reduced ALDH activity. (A) Levels of acetaldehyde in adult males exposed to 100% ethanol. (n = 4). (B) *CrzR^01^* mutation resulted in the reduction of whole cell ALDH activity (n = 3). (C) Succinate dehydrogenase activity in mitochondria (Mt) and cytosol (Ct). The activity was detected almost exclusively in the mitochondrial fraction (n = 3). (D) Reduction of mitochondrial ALDH activity (AL) in *CrzR^01^* mutant. Succinate dehydrogenase activity (SD) is similar between control and mutant. (n = 3). (E, F) Crz-CD leads to reduced ALDH activity. (E) Whole cell ALDH activities of *Crz::hid* are significantly lower than those of the transgenic controls (n = 8). (F) *grim*-induced Crz-CD produced significant reduction of mitochondrial ALDH activity (n = 3). (*P<0.05; **P<0.01; ***P<0.001; ns, not significant). Each data point represents mean ± sem for the indicated replicates. All genotypes are in *y w* background.

Ethanol is first oxidized to acetaldehyde, which has been considered a major cause of hangover symptoms in humans [32]. To test this possibility for *CrzR^01^* flies, we used gas chromatography to monitor the contents of acetaldehyde following exposure to ethanol. Remarkably acetaldehyde contents in both *CrzR^01^* and *CrzR^01^/Df* flies were up to 100% higher than those of control flies 45 min after ethanol exposure (Figure 4A).

The accumulation of acetaldehyde in *CrzR^01^* mutants is possibly due to subnormal acetaldehyde oxidation as a result from lower ALDH activity. Indeed, ALDH activity in whole fly extract of *CrzR^01^* was only 63% of wild-type (Figure 4B). Since acetaldehyde oxidation occurs mainly inside the mitochondria, the ALDH activity was measured with isolated mitochondria. Succinate dehydrogenase activity, a mitochondrial marker, was observed almost exclusively in the mitochondrial fraction in both genotypes, verifying successful separation of the mitochondria from the cytosol (Figure 4C). Around 30% reduced ALDH activity was observed in the *CrzR^01^* mutant, while succinate dehydrogenase activity was indistinguishable between wild-type and *CrzR^01^* (Figure 4D). Reduction of ALDH activity was also observed in whole cell extracts of Crz-CD flies (*Crz::hid*) or in the mitochondrial fraction of Crz-CD induced by ectopic *grim* expression (Figure 4, E and F). In aggregate, these results suggest that Crz signaling plays an important role in the regulation of ALDH activity.

### Loss of CrzR results in enhanced ADH activity

**Figure 5:**
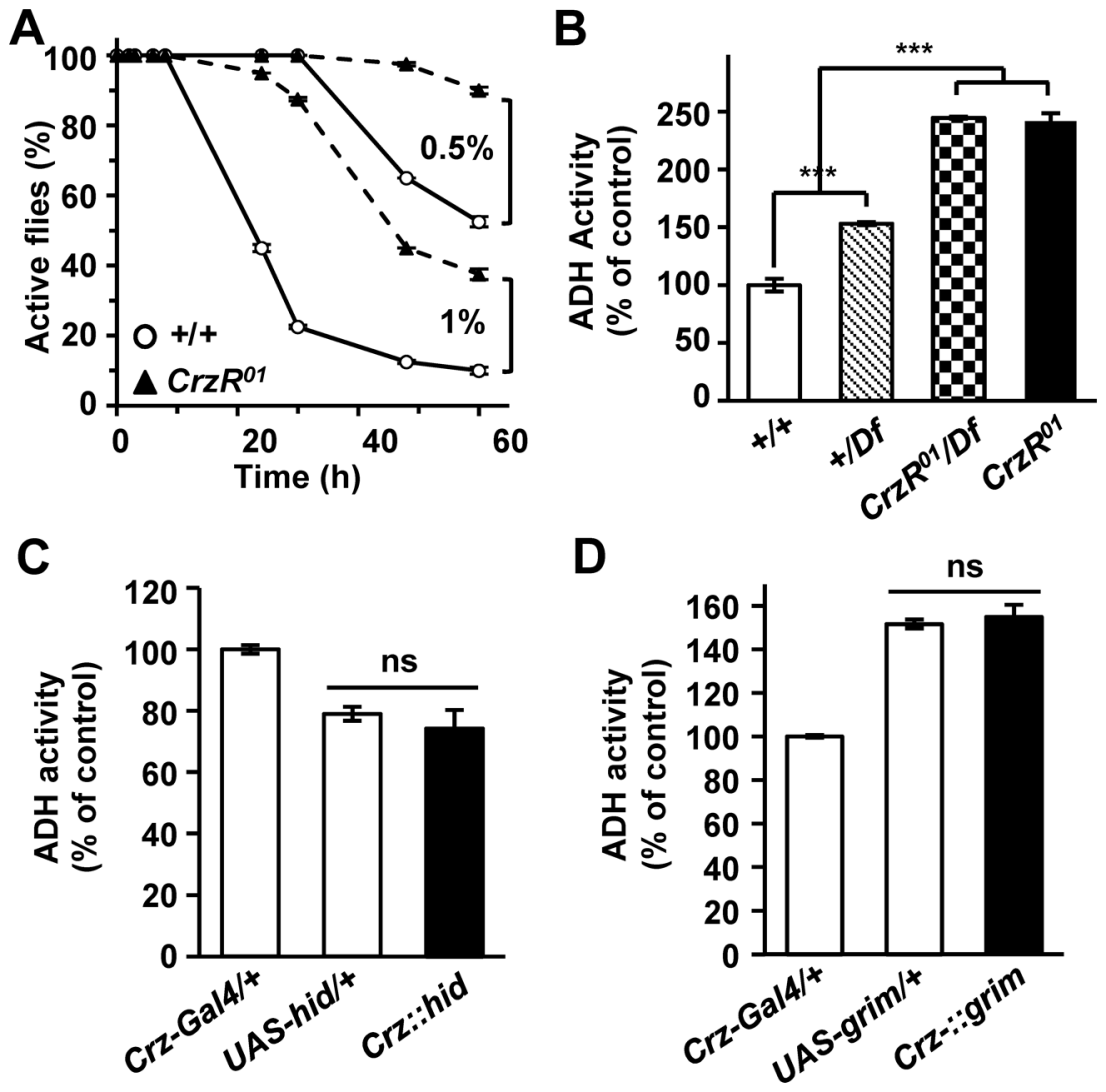
*CrzR* mutation causes significant increase of ADH activity. (A) Survival rates of *CrzR^01^* (dashed lines) and wild-type (solid line) in response to acetaldehyde as indicated (n = 3). (B) Histogram showing ADH activity (n = 4). (C,D) ADH activity in Crz-CD. (C) *hid-* and (D) *grim*-induced Crz cell ablation did not increase the whole cell ADH activity (*P* > 0.05, n  =  3). (*** *P*<0.001). Each data point represents mean ± sem for the indicated replicates. All genotypes are in *y w* background.

Since *CrzR^01^* mutants have lower ALDH activity, they could be more sensitive to exogenously provided acetaldehyde than wild-type. To test this, flies were fed low concentrations of acetaldehyde without causing acute toxicity [33]. Surprisingly, the *CrzR^01^* flies were more resistant to acetaldehyde-mediated intoxication than the wild-type. With 0.5% (v/v) acetaldehyde, ∼ 50% of wild-type flies became immobilized after 60 h, while only 10% of *CrzR^01^* did so (Figure 5A). Significant differences between the two genotypes were also apparent in response to 1% acetaldehyde.

Since *D. melanogaster* ADH is known to mediate the detoxification of the acetaldehyde by converting acetaldehyde to ethanol [33], we wondered if greater resistance to acetaldehyde by *CrzR^01^* is due to higher ADH activity in the mutant. Indeed, around 2.4-fold increased ADH activity was measured in both homozygous *CrzR^01^* and *CrzR^01^*/*Df* as compared to wild-type (Figure 5B). *Df/+* flies showed a 1.5-fold increase of ADH activity, suggesting a dosage-dependent regulation of the ADH by CrzR.

In contrast to *CrzR^01^*, Crz-CD did not elevate ADH activity (Figure 5, C and D), indicating that *Crz* neurons are required only for the regulation of ALDH activity. The reasons for such difference are currently unclear but may be related to an unknown ligand that controls CrzR activity or intrinsic activity of CrzR (see discussion).

### CrzR regulates Adh transcription in PKA-dependent manner

**Figure 6:**
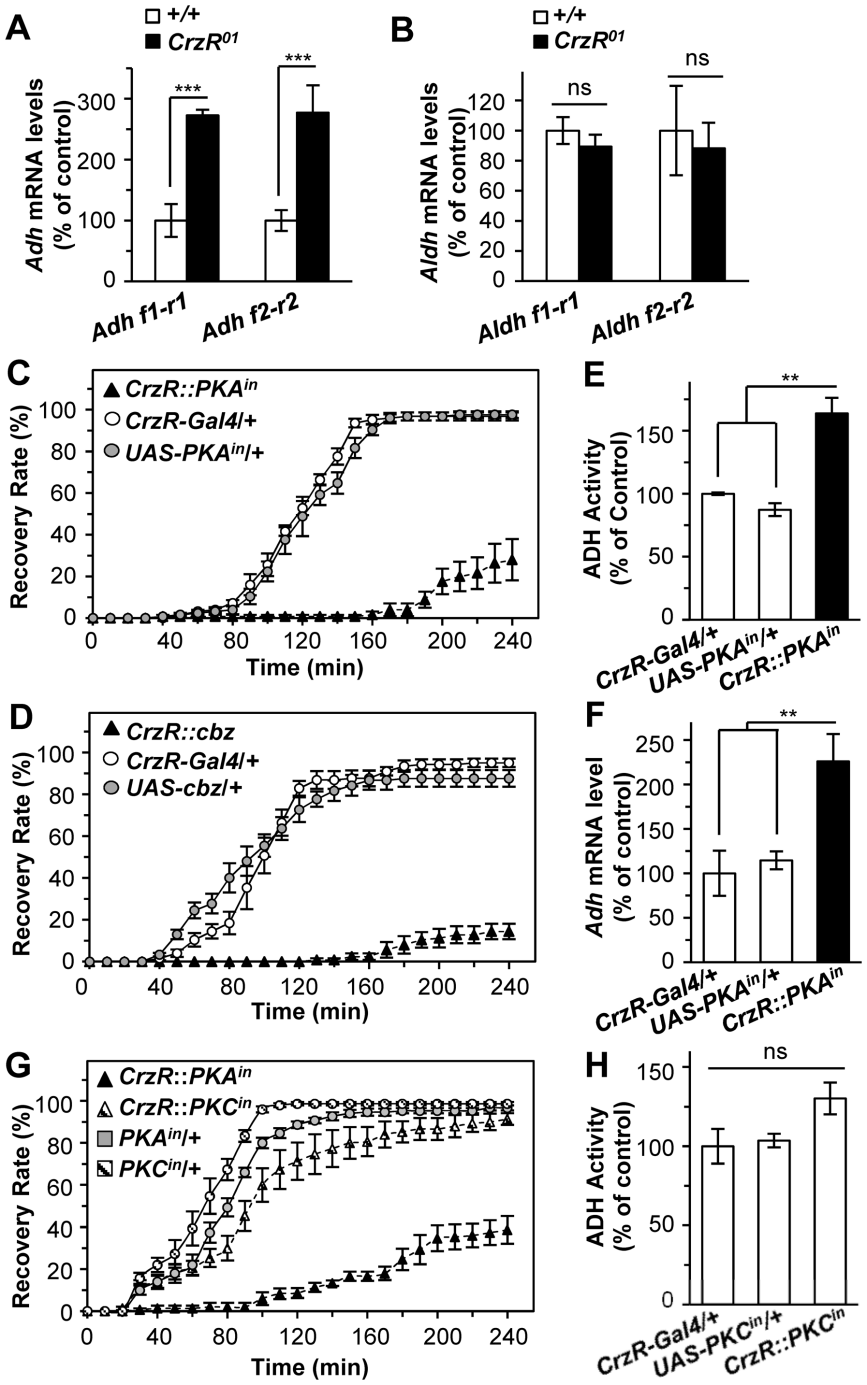
CrzR regulates *Adh* mRNA levels through PKA-dependent pathway. (A) RT-qPCR using two different sets of primers revealed 2.7-fold increase of *Adh* transcript level in *CrzR^01^* compared to the wild-type (n = 4). (B) No significant change of *Aldh* transcript level in *CrzR^01^* (n = 4). (C,D) Expression of *PKA* inhibitor (C) or dominant negative CREB (D) using *CrzR-gal4* induced severe hangover-like phenotype (n = 5). (E) PKA inhibition in CrzR cells increased ADH activity by 50% compared to the controls (n = 3). (F) PKA inhibition in CrzR cells leads to significant increase of *Adh* transcript level (n = 3). (G) No delayed recovery was observed from flies expressing PKC inhibitor from *CrzR-Gal4* (*P* > 0.05, n  =  5). (H) Inhibition of PKC did not affect ADH activity (*p* > 0.05, n  =  3). (**P<0.005; *** *P*<0.001). Each data point represents mean ± sem for the indicated replicates. All genotypes are in *y w* background.

To further delve into the mechanisms as to how Crz/CrzR regulates ADH and ALDH activities, mRNA levels of *Adh* and *Aldh* were analyzed by real-time RT-PCR. An average of 2.7-fold increase of *Adh* transcript levels were observed from the *CrzR^01^* using two different primer sets (Figure 6A) while no difference was found for *Aldh* (Figure 6B). These results indicate that CrzR is involved in the transcriptional regulation of *Adh*, but not of *Aldh*.

**Figure 7:**
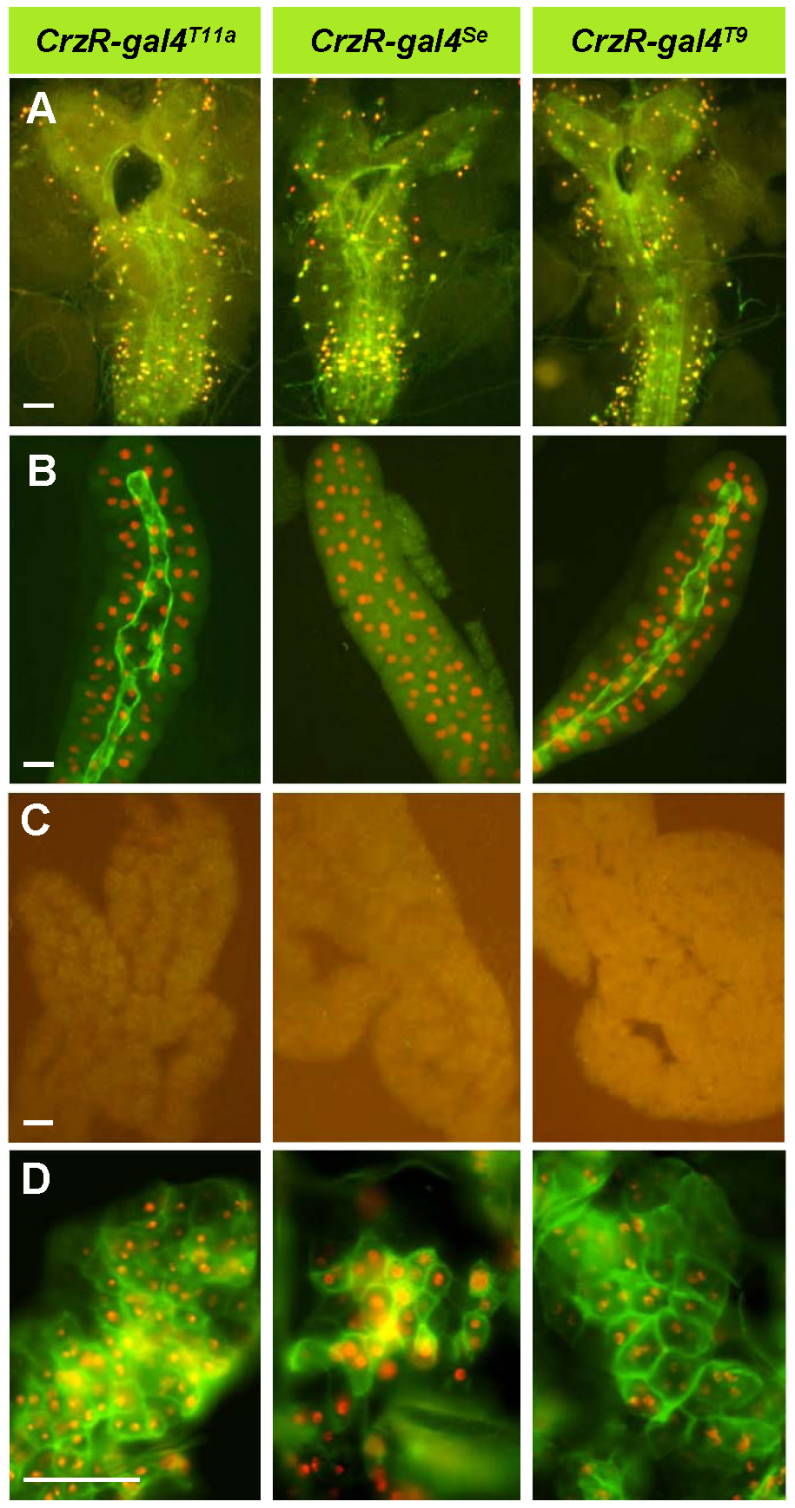
*CrzR-gal4* driven reporter gene expression. *UAS-mCD8GFP*;; *UAS-Redstinger* was expressed under the control of three *CrzR-gal4* drivers as indicated. (A-C) Third instar larva. (A) CNS. (B) salivary gland. (C) fat body. (D) Adult fat tissue. Scale bar = 50 µm. (green, membrane bound GFP; red, nuclear RFP).

To elucidate signaling mechanisms underlying CrzR-regulated *Adh* transcription, transgenic manipulations were performed in the CrzR cells using a *CrzR-gal4* line. Although we have been unsuccessful to obtain endogenous *CrzR* expression patterns using *in situ* hybridization, reporter gene expression patterns driven by the *CrzR-gal4* lines were consistent with high throughput expression data from FlyAtlas (Flybase: flybase.org; see also ref. 28). Simultaneous expression of membrane-bound GFP (*mCD8GFP*) and nuclear RFP (*Redstinge*r) showed respective florescent signals in the larval central nerve system (CNS) and salivary glands, but not in the larval fat body (Figure 7 A-C). Interestingly, unlike in the larval fat body, strong fluorescent signals were detected in the adult fat body, indicating developmental regulation of *CrzR* expression in this metabolic tissue (Figure 7D). Such spatial and developmental expression patterns agree with the FlyAtlas data. Moreover, since the fat body is the major target tissue for the neuroendocrine regulation of hemolymph trehalose levels [34] and Crz is likely to be involved in such physiological event [25], *CrzR* expression in the adult fat body was expected.

CrzR is a member of Class-A GPCR family [20], [29], [35]. A signaling pathway typical of these receptors involves PKA, which regulates activity of a transcription factor CREB via phosphorylation. Thus we explored whether this is true for the CrzR-regulated expression of *Adh*. To do this, PKA signaling was disrupted by ectopic expression of a PKA inhibitor (*PKA^in^*), a dominant-negative form of the regulatory subunit of a *Drosophila* PKA [36], or Cbz, a dominant negative CREB [37] using *CrzR-gal4*. Remarkably, hangover-like phenotypes of these flies were comparable to that of the *CrzR^01^* mutant (Figure 6, C and D). In addition, the inhibition of PKA also increased ADH activity and *Adh* mRNA levels significantly (Figure 6, E and F), suggesting that *CrzR-*regulated *Adh* expression requires PKA-dependent pathway. In contrast, ectopic expression of the protein kinase C (PKC) inhibitor [38] showed a wild-type pattern of the hangover-like phenotype and ADH activity (Figure 6, G and H), indicating no positive role for PKC.

## Discussion

Drinking alcohol is one of the oldest habits of humankind and has some positive effects on human society, but uncontrolled consumption causes serious psychopathic symptoms and other health problems. Excessive consumption of alcoholic beverage and associated hangovers cost $223.5 billion per year in the U.S. alone, due to losses in workplace productivity, health care expenses, costs associated with law enforcement and criminal justice, and accidents (http://www.cdc.gov/Features/AlcoholConsumption/).

Depending on the concentrations, effects of ethanol consumption in humans include euphoria, impaired motor function and speech, followed by vomiting, coma and even death in certain cases. Upon cessation of drinking, hangover is characterized by unpleasant physical pains such as headache, sensory problems such as vertigo, gastrointestinal symptoms such as nausea and vomiting, and disruption of sleep and biological rhythms [39]. These symptoms are mainly from acetaldehyde accumulation, as supported by high incidence of ethanol intoxication in Eastern Asian populations due to the polymorphic deficiency of functional *ALDH2* allele [40], [41] and *ALDH2*-knockout mice [42]. In line with this, drugs inhibiting ALDH, such as disulfiram, have been used to treat chronic alcoholism by causing adverse symptoms from ethanol intake [43], [44]. Despite these reports, relatively little is known about the regulation of ethanol metabolism by physiological factors.

Like mammals, *D. melanogaster* metabolizes ethanol to acetaldehyde and acetate catalyzed by ADH and ALDH, respectively [45], and *Adh* and *Aldh* mutant flies showed dramatically reduced tolerance to ethanol [46]. Intriguingly, our present data suggest that a neuroendocrine system involving Crz plays an important role in regulating ADH and ALDH activities. CrzR activation leads to the PKA-dependent transcriptional repression of *Adh* and to post-transcriptional activation of ALDH via unknown pathways, which together suppress acetaldehyde accumulation.

In contrast to *CrzR^01^*, Crz-CD did not affect ADH activity levels, but reduced ALDH activity. Such a difference could explain that the Crz-CD flies displayed milder hangover-like phenotype than *CrzR^01^* did. One might argue that Crz-CD still has residual Crz function. Although this is a possibility, undetectable Crz neurosecretory cells in the Crz-CD brains indicate near lack of Crz function. This is much more severe than RNAi-induced *Crz* knockdown with respect to the level of *Crz* expression [16]. Another caveat is that Crz-CD phenotype is due to an elimination of other co-existing transmitters. In fact, a subset of Crz neurons was shown to co-express small neuropeptide F (sNPF) [47]. However, since sNPF-producing neurons are so widely distributed in the brain, ablation of a few neurons co-expressing Crz and sNPF is unlikely to affect sNPF functions. Although more definitive evidence for the Crz await *Crz*-null mutant, our data with *CrzR*-null mutant support a role for Crz in the hangover-like phenotype.

Assuming that Crz-CD eliminates Crz function nearly entirely, we propose that CrzR mediates two separate signaling pathways; Crz-dependent up-regulation of ALDH activity and Crz-independent down-regulation of *Adh* transcription. The latter pathway might involve a distinct ligand that activates CrzR. One possible candidate is adipokinetic hormone (AKH), as the AKH receptor is structurally related to the CrzR with 56% amino acid sequence similarity [29]. However, CrzR showed very little affinity for AKH [35], indicating that AKH is unlikely to be a natural ligand for CrzR. Alternatively, CrzR might have an intrinsic activity that is not required for ligand binding. It is not uncommon that some GPCRs have intrinsic (or spontaneous) activities in the absence of ligand biding [48].

Although the identity of the second ligand for CrzR is speculative, a growing body of evidence suggests that a GPCR couples to different G-proteins in response to different agonists; such molecular flexibility is often referred to as “functional selectivity” of GPCR [49], [50]. According to this hypothesis, binding properties of different ligands induce and stabilize a unique conformational status of GPCR, which in turn shifts coupling preference to different G-proteins. Recently, CrzR isolated from the silkworm, *Bombyx mori*, was shown to couple dually to the Gq and Gs proteins in cell-based assays [51]. However, our results indicate that Gq-associated PKC activation is unlikely to be involved in the regulation of *Adh* transcription in *Drosophila*. Thus we propose that Gs-led PKA activation is the major *in vivo* signaling pathway of the CrzR at least for the *Adh* regulation. Nevertheless, it seems that CrzR is an excellent model system to unravel the physiological dynamics of the GPCR.

The International Agency for Research on Cancer (IARC) classified ethanol as a Group 1 carcinogen (http://monographs.iarc.fr/ENG/Classification/ClassificationsAlphaOrder.pdf). Accumulation of acetaldehyde as a result of defective ALDH2 is a major culprit of the carcinogenesis [52], [53]. In line with this, many cancer cells have disproportional activities of ADH and ALDH, making them less efficient in removing acetaldehyde compared to normal tissues [54]. In this regard, *CrzR^01^* flies might be prone to cancer development, and perhaps can serve as an interesting model system to elucidate the mechanisms of ethanol-induced carcinogenesis.

## Materials and Methods

### Fly strains

Flies were reared on standard cornmeal/yeast medium at 25°C. The following strains were used: *y w*; *Mi{ET1}GRHRII^MB00838^* (Bloomington stock no. 22910), *w^1118^*; *sna^Sco^/SM6a, P{hsILMiT}2.4* (Bloomington stock no. 24613), *w^1118^*; *UAS-PKA^in^* (gifted from Ben White at NIH), *w^1118^*; *UAS-PKC^in^* (Bloomington stock no. 4589), *y w*;; *UAS-cbz/TM3, Sb, Ser* (Bloomington stock no. 7222). *w^1118^;; Df(3L)BSC380/TM6C, Sb^1^* (for short *Df;* Bloomington stock no. 24404). The following transgenic lines were used to induce targeted ablation of Crz neurons: *y w;; Crz-gal4* [25], *y w; UAS-rpr, y w;; UAS-▵rpr, y w;; UAS-grim,* and *y w; UAS-hid/CyO, y^+^* [55].

### *CrzR* null mutant and *CrzR-gal4* line

Mobilization of the *y w*; *Mi{ET1}GRHRII^MB00838^* (Bloomington stock no. 22910) was used to induce *CrzR* mutation. Homozygous *GRHRII^MB00838^* females were crossed to males carrying a genomic source of heat-shock inducible *Minos* transposase (*hsLMit^2.4^*). Progenies were incubated at 37°C water bath for 1 h per day until pupariation. Fifty single male progeny with eyes showing mosaic pattern of GFP were crossed individually to a balancer *y w*;; *Ly/TM6C, Sb, Tb* females. Two GFP-negative progeny derived from each cross were randomly selected and crossed individually to the balancer to establish a total of 100 excision lines. Genomic DNA from homozygous flies was used for PCR-based screening to detect the deletion of the *CrzR* locus. For *CrzR-gal4*, 3.5-kb sequence upstream of the *CrzR* (–3130 to +331, +1 is transcription start site) was PCR amplified (primer sequences are shown in Table S1). This fragment was cloned into the pPTGAL vector at Xba I/Not I sites for germline transformation.

### Behavior assays

Groups of 25 males (1–3 days old) were maintained in a food vial for 1–2 days and used for the following behavior assays.

**Recovery from ethanol sedation.** Flies were transferred into an empty plastic vial (O.D.×H: 25×95 mm) and then sealed with cotton plug. One ml of 100% ethanol was applied onto the cotton plug. Following 17–18 min of exposure, the cottons were replaced with fresh buzz plugs and then the vials were placed upside down. Flies capable of climbing up were considered “recovered” and the numbers of recovered flies were recorded every 10 min.

**Toxicity assay.** Assay was performed according to Barbancho *et al* [56], with slight changes. Groups of 20 males were placed in hermetically sealed plastic vials (O.D.× H: 25×95 mm) containing 3% sucrose medium supplemented with acetaldehyde. The toxicity of acetaldehyde was represented by the number of knocked-down flies as a function of exposure time.

Procedures of fecundity, circadian rhythm and ethyl ether recovery assays are provided in supporting protocol (Protocol S1).

### Activity assays of ADH and ALDH

A hundred flies were homogenized in 1 ml of isolation buffer [50 mM sodium phosphate (pH 7.4), 0.24 M sucrose, 0.5 mM EDTA, 0.5 mM dithiothreitol, 0.001% (w/v) phenylthiourea], as described [57]. The homogenate was centrifuged for 10 min at 1000 rpm and then the supernatant was saved. The pellet was resuspended in 0.5 ml of the isolation buffer. Following centrifugation as before, the supernatant was combined with the first one. The pooled supernatant was centrifuged for 45 min at 14,000 rpm to separate cytosolic and mitochondrial fractions [58]. The pellet was washed with isolation buffer twice and resuspended in 250 µl of the same buffer supplemented with 1% (v/v) Triton-X-100. The resuspension was allowed to stand for 15 min and then centrifuged at 14,000 rpm for 20 min. The supernatant was used for mitochondrial fraction. All operations were performed on ice except centrifugation at 4°C. The protein content was determined by the BCA (Bicinchoninic acid) method. Three independent extracts per genotype were prepared. ALDH and ADH activities were assayed spectrophotometrically by measuring reduced β-NAD^+^ at 340 nm. ALDH activity assay was done following the method of Moxon *et al.* [59]. Briefly, the assay was started by adding 25 µl fly extract into 500 µl of 50 mM Tris/HCl (pH 8.6) including 3.6 mM acetaldehyde, 1 mM β-NAD^+^, and 20 mM pyrazole. The absorbance was recorded every min for up to 20 min by using Smartspect™ 3000 kinetics program (BioRad). ADH activity was assayed, as described in Barbancho *et al.* [56], by adding 20 µl extract into 500 µl of 50 mM Tris/HCl (pH 8.6) buffer containing 100 mM 2-propanol, 1 mM β-NAD^+^ and 1.6 mM cyanamide. The absorbance was recorded every 10 sec for up to 3 min. Succinate dehydrogenase activity was assayed by following the reduction of INT [2-(p-indophenyl)-3-(p-nitrophenyl)-5-phenyl tetrazolium] [60]. The reaction was initiated by adding 2 µl of mitochondrial or 20 µl of cytosolic fraction into 200 µl substrate solution (0.11% w/v INT, 55 mM sodium succinate, 25 mM sucrose in 100 mM sodium phosphate, PH 7.4), followed by incubation in 37°C water bath for 15 min. The reaction was stopped by adding 200 µl of 3% ice-cold HCl. The chromogen was extracted with 600 µl ethyl acetate by centrifugation at 14,000 rpm for 5 min, and then its absorption was determined at 490 nm.

### Measurement of acetaldehyde content

Forty flies were homogenized in 1 ml sterilized ice-cold water with 20 mM pyrazole and 1.6 mM cyanamide to inhibit ADH and ALDH activities. The homogenates were centrifuged at 14,000 rpm for 20 min at 4°C and the supernatant was kept in ice. Standard solutions were prepared from pure acetaldehyde in the same solution. Acetaldehyde content was measured using an Agilent 7890 gas chromatograph equipped with Agilent model G1888 headspace auto sampler, a flame ionization detector and a DB-624 capillary column (60 m x 0.32 mm x 1.8 µm). Separation of acetaldehyde was complete under the following conditions; injection volume 0.2 µl; split ratio 50:1; injection port temperature 200°C; oven temperature program: 60°C for 2 min, 25°C min^−1^ to 200°C for 1 min, FID temperature 300°C; carrier gas N_2_; and gas flow 3.0 ml/min (helium). For the auto sampler: auto sampler oven temperature 70°C; transfer line temperature 125°C; loop temperature 125°C; vial equilibration time 15 min; high shaking (mixing) speed; loop fill time 0.03 min; inject time 0.50 min; vial pressure 10 psig; pressurization time 0.5 min. All determinations were carried out in triplicates.

### Quantitative RT-PCR

Total RNA was purified from 20 adult males using TRIzol reagent (Invitrogen) according to the manufacture’s protocol. About 5–10 µg of total RNA was added to 40 µl of Go Superscript reverse transcription mix (Promega) to generate cDNA using the oligo-dT-Ad primer (GACTCGAGTCGACATCGAT_20_). cDNA was then purified and eluted with 40-μl water using a PCR purification kit (Qiagen). SYBR Green reaction kit (Bio-Rad) was used for real-time PCR, with 0.5 µl cDNA included in 25 µl reaction volume. The PCR was carried out using an iQ™5 Multicolor Real-Time PCR Detection System (Bio-Rad). Amplifications were initiated with a 10-min denaturation at 95°C, followed by 40 cycles of 95°C, 15sec 55°C, 30 sec 72°C, 30 sec. Reactions were run in triplicates. Primers (Table S1) were designed to flank at least one intron to ascertain that the PCR products were derived from the cDNA. β-*tubulin* was used as an internal control to normalize expression levels of the target genes. Expression data were obtained using the 2^−ΔΔCT^ method, as described by Livak and Schmittgen [61] where ΔΔ*CT* equals the normalized cycle threshold (Δ*CT*) of *Aldh* or *Adh* in test genotypes minus the Δ*CT* of the same gene in control flies.

### Histology

*CrzR-gal4* line was crossed to *UAS-mCD8GFP*;; *UAS-Redstinger.* The resulting male offspring were dissected and incubated in fixative (4% paraformaldehyde in PBS) for 30 min at room temperature. Tissues were washed in PBS (3×5 min) and mounted in a quenching medium (0.5% n-propyl gallate in 90% glycerol and 10 mM phosphate buffer, pH 7.4). Differential fluorescent signals were captured by Olympus BX-61 microscope equipped with a CCD camera and then merged.

### Statistics

Statistical analyses were done using Instat 2.0 (GraphPad Software). Student’s unpaired *t*-test was used to determine significant differences between two means. Difference among multiple means was tested by one-way ANOVA followed by Student-Newman-Keuls (SNK) multiple comparisons test.

## Supporting Information

Figure S1 **Fecundity assay**. Numbers of eggs laid per fly were recorded per day (n = 8). No difference was found between *CrzR^01^* and wild type females.
(PDF)

Figure S2 **Actograms showing circadian locomotor activity rhythms.** Flies were entrained for 3 days of 12:12 LD followed by 7 days of DD. A majority of *CrzR^01^*/*Df* (23/25; 92%) and *Df*/*+*(26/27; 96%) flies showed normal circadian rhythmic activities, with the mean period length (± sem) of 23.9 h (± 0.05) for *CrzR^01^*/*Df* and 23.7 h (± 0.07) for *Df*/*+*.
(PDF)

Figure S3 **Knocking down of** ***CrzR*** **mRNA leads to severe hangover-like phenotype.** (A) RT-PCR showed significant reduction of *CrzR* mRNA levels using two *UAS-CrzR^RNAi^* lines, S3S and T8. (B) Inducing *CrzR^RNAi^* with *actin-gal4* driver (triangles) reuslted in severe hangover-like phynotype, compared to transgenic controls (circles). Each data point is a mean ± sem (n = 5). All genotypes are in *y w* background.
(PDF)

Figure S4 **Recovery test from ethyl ether-induced sedation.** (A) No obvious difference of the recovery rate was observed between Crz-CD flies (triangles) and controls (squares). Each data point represents mean ± sem (n = 3). (B) No delayed recovery was observed for *CrzR^01^* mutant (n = 4).
(PDF)

Table S1 **Primers used.**
(PPTX)

Protocol S1 **Supporting protocol.**
(DOCX)
