# Supplementary figures and images for: Regulation of Ethanol-Related Behavior and Ethanol Metabolism by the Corazonin Neurons and Corazonin Receptor in Drosophila melanogaster
Source: PLoS One. 2014 Jan 28;9(1):e87062. doi: 10.1371/journal.pone.0087062 (PMC3904974; doi:10.1371/journal.pone.0087062)

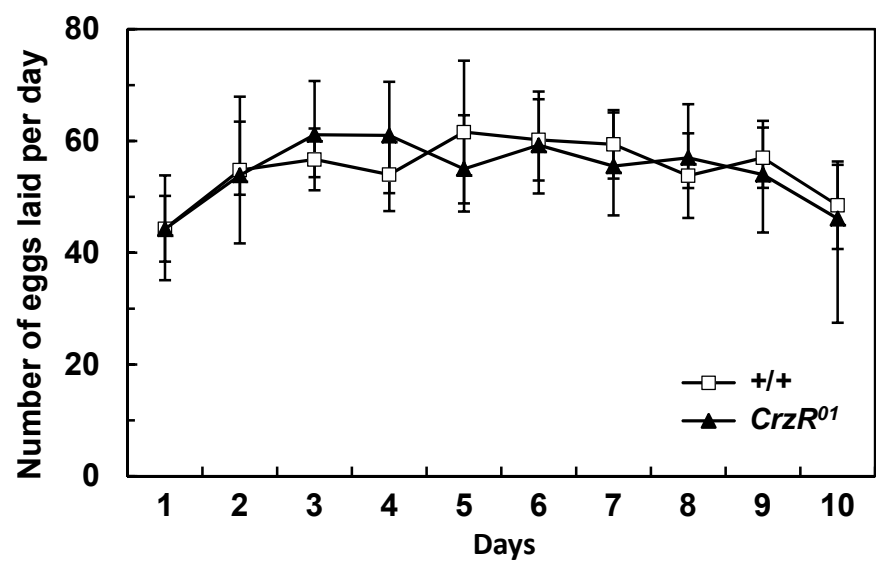

Supplement: Figure S1 — Fecundity assay. Numbers of eggs laid per fly were recorded per day (n = 8). No difference was found between CrzR01 and wild type females. (PDF) [file pone.0087062.s001.pdf]

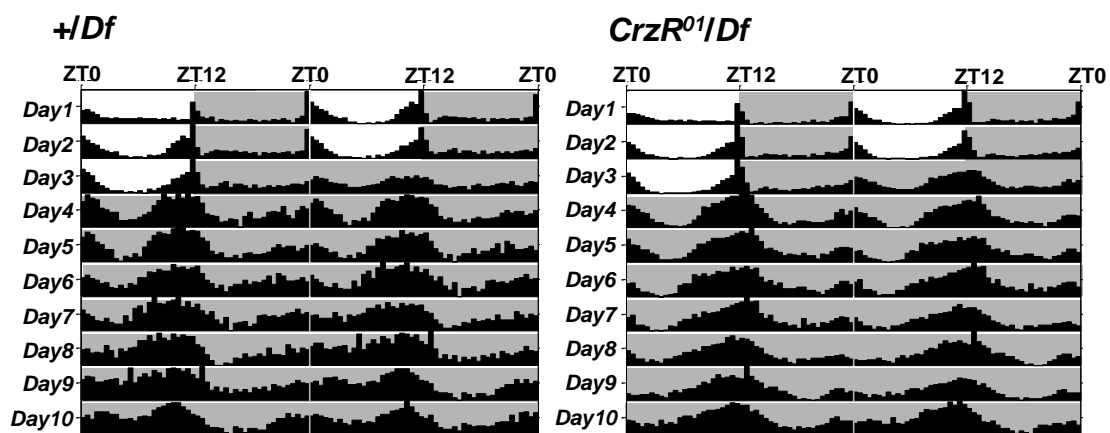

Supplement: Figure S2 — Actograms showing circadian locomotor activity rhythms. Flies were entrained for 3 days of 12:12 LD followed by 7 days of DD. A majority of CrzR01/Df (23/25; 92%) and Df/+(26/27; 96%) flies showed normal circadian rhythmic activities, with the mean period length (± sem) of 23.9 h (± 0.05) for CrzR01/Df and 23.7 h (± 0.07) for Df/+. (PDF) [file pone.0087062.s002.pdf]

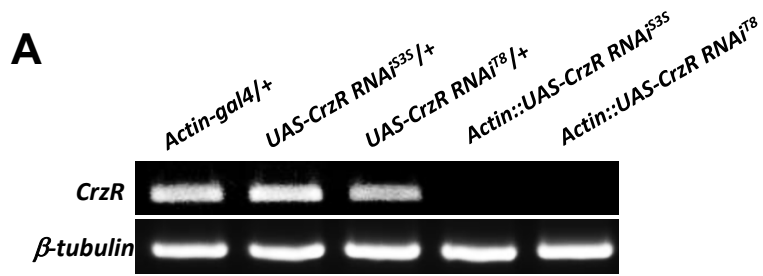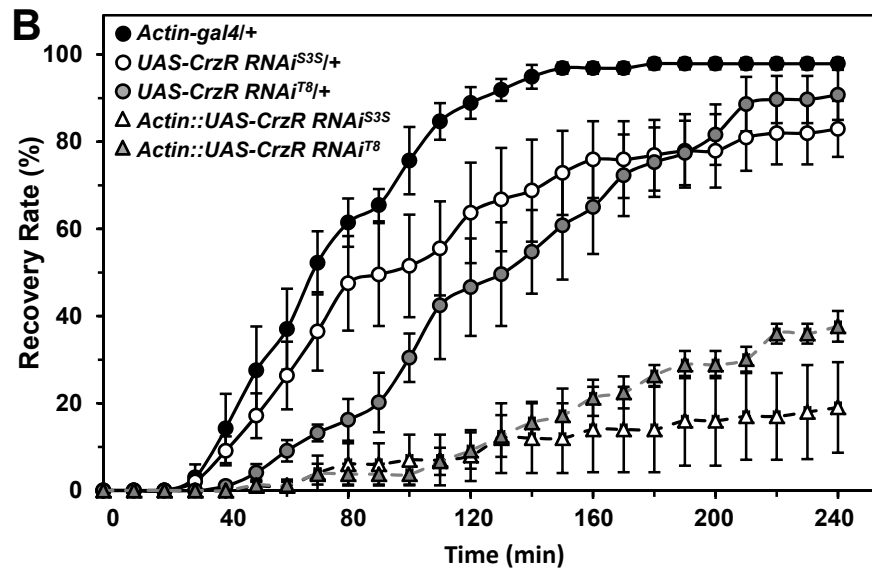

Supplement: Figure S3 — Knocking down of CrzR mRNA leads to severe hangover-like phenotype. (A) RT-PCR showed significant reduction of CrzR mRNA levels using two UAS-CrzRRNAi lines, S3S and T8. (B) Inducing CrzRRNAi with actin-gal4 driver (triangles) reuslted in severe hangover-like phynotype, compared to transgenic controls (circles). Each data point is a mean ± sem (n = 5). All genotypes are in y w background. (PDF) [file pone.0087062.s003.pdf]

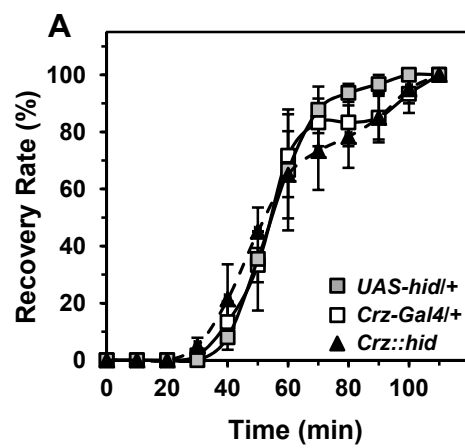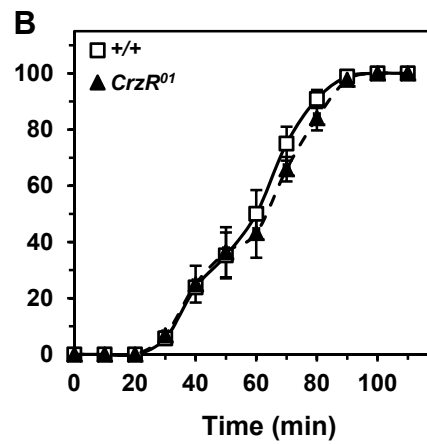

Supplement: Figure S4 — Recovery test from ethyl ether-induced sedation. (A) No obvious difference of the recovery rate was observed between Crz-CD flies (triangles) and controls (squares). Each data point represents mean ± sem (n = 3). (B) No delayed recovery was observed for CrzR01 mutant (n = 4). (PDF) [file pone.0087062.s004.pdf]
