# Supplementary material for: Regulation of Ethanol-Related Behavior and Ethanol Metabolism by the Corazonin Neurons and Corazonin Receptor in Drosophila melanogaster
Source: PLoS One. 2014 Jan 28;9(1):e87062. doi: 10.1371/journal.pone.0087062 (PMC3904974; doi:10.1371/journal.pone.0087062)
Supplement: Table S1 — Primers used. (PPTX) [file pone.0087062.s005.pptx]

## Slide 1
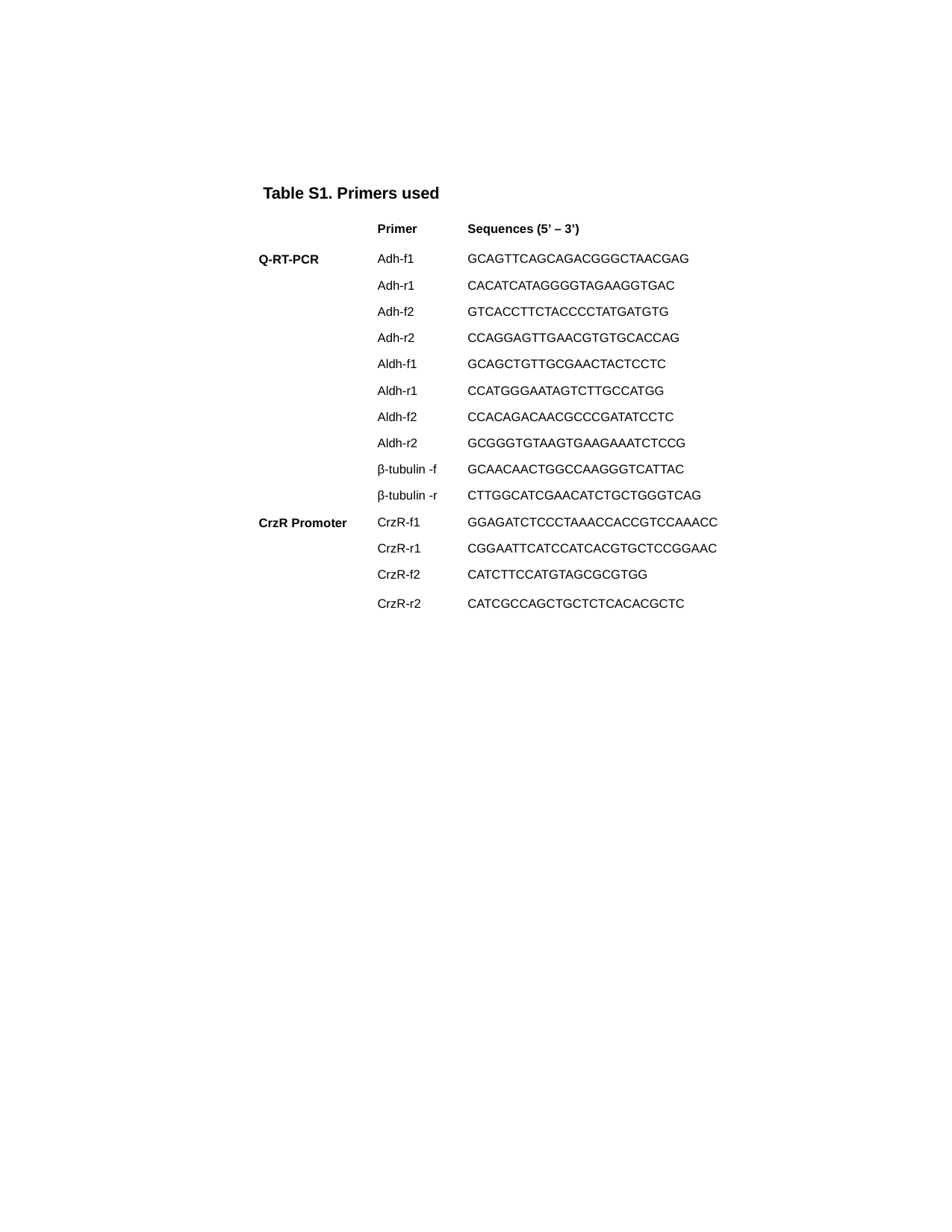

Table S1. Primers used
| | Primer | Sequences (5’ – 3’) |
| --- | --- | --- |
| Q-RT-PCR | Adh-f1 | GCAGTTCAGCAGACGGGCTAACGAG |
| | Adh-r1 | CACATCATAGGGGTAGAAGGTGAC |
| | Adh-f2 | GTCACCTTCTACCCCTATGATGTG |
| | Adh-r2 | CCAGGAGTTGAACGTGTGCACCAG |
| | Aldh-f1 | GCAGCTGTTGCGAACTACTCCTC |
| | Aldh-r1 | CCATGGGAATAGTCTTGCCATGG |
| | Aldh-f2 | CCACAGACAACGCCCGATATCCTC |
| | Aldh-r2 | GCGGGTGTAAGTGAAGAAATCTCCG |
| | β-tubulin -f | GCAACAACTGGCCAAGGGTCATTAC |
| | β-tubulin -r | CTTGGCATCGAACATCTGCTGGGTCAG |
| CrzR Promoter | CrzR-f1 | GGAGATCTCCCTAAACCACCGTCCAAACC |
| | CrzR-r1 | CGGAATTCATCCATCACGTGCTCCGGAAC |
| | CrzR-f2 | CATCTTCCATGTAGCGCGTGG |
| | CrzR-r2 | CATCGCCAGCTGCTCTCACACGCTC |
