# Supplementary material for: Regulation of Ethanol-Related Behavior and Ethanol Metabolism by the Corazonin Neurons and Corazonin Receptor in Drosophila melanogaster
Source: PLoS One. 2014 Jan 28;9(1):e87062. doi: 10.1371/journal.pone.0087062 (PMC3904974; doi:10.1371/journal.pone.0087062)
Supplement: Protocol S1 — Supporting protocol. (DOCX) [file pone.0087062.s006.docx]

**Supporting protocol**

**Recovery from ethyl ether-induced sedation**

Groups of twenty-five males (1-3 days old) were maintained in a food vial for 1-2 days and used for the following behavior assays. Flies were placed into an empty plastic vials, as described above. Fifty μl of pure ethyl ether was applied onto the cotton plug. Following 10 min of exposure, the cottons were replaced with fresh buzz plugs and then the vials were placed upside down. The numbers of recovered flies were recorded every 10 min.

**Fecundity Assay**

A single wild-type or *CrzR^01^* virgin female was crossed to two wild-type males, and kept in a food vial for 24 h before assay started. The flies were reared in fresh food vials with dry yeast powder to allow them to lay eggs for 24 h before transferred to new food vials. The numbers of eggs laid in each vial were recorded every 24 h for up to 10 days. All food vials were kept in a humidified chamber at around 25 °C.

**Circadian rhythms**

Males were entrained to 3 days of 12-h light: 12-h dark cycles (LD), and then preceded into constant darkness (DD) conditions for 7 days. Locomotor activities of individual fly were monitored using activity monitors, and the data were analyzed using ClockLab software (Actimetrics) as described previously [[1](#_ENREF_1)].

**CrzR RNAi construct**

*CrzR* cDNA from -59 to +328 (+1 indicates start codon) was PCR amplified (primer sequence in are shown in Table S1). The PCR product was subcloned into *sympUAS* vector at *Bgl* II/ *Eco*R I sites for germ-line transformation [2]. The efficiency of *CrzR^RNAi^* was tested by RT-PCR using a universal driver *actin-Gal4*. Total RNA from 20 adults was purified using TRIzol reagent (Invitrogen) according to the manufacture’s protocols and then 500 ng of the RNA was added to 25 μl of Superscript III one-step RT-PCR mix (Invtrogen).

**References**

1. Bahn JH, Lee G, Park JH (2009) Comparative analysis of Pdf-mediated circadian behaviors between *Drosophila melanogaster* and *D. virilis*. Genetics 181: 965-975.

2. Giodano E, Rendina R, Peluso I, Furia M (2002) RNAi triggered by symmetrically transcribed transgenes in *Drosophila melanogaster*. Genetics 160: 637-648.
